# Local atomic environments reveal structural controls on MgSiO_3_ liquid-crystal similarity

**DOI:** 10.1093/pnasnexus/pgag347

**Published:** 2026-10-06

**Authors:** Jake Acquadro, Xiyuan Bao, Jie Deng

**Affiliations:** Department of Geosciences, Princeton University, 11 Ivy Lane, Princeton, NJ 08540, USA; Department of Earth and Planetary Sciences, Harvard University, 20 Oxford Street, Cambridge, MA 02138, USA; Department of Geosciences, Princeton University, 11 Ivy Lane, Princeton, NJ 08540, USA

**Keywords:** SOAP, principal component analysis, mineralogy, perovskite, MgSiO_3_

## Abstract

MgSiO_3_ is a fundamental silicate system in Earth and rocky planetary interiors, where its structures control crystallization, differentiation, and evolution of host planets. Yet, silicate structure has usually been analyzed through aggregate structural observables that do not place individual local atomic environments from crystals and liquids into a common similarity space. Here, we compare local environments across eight MgSiO_3_ polymorphs and the liquid up to 700 GPa and 12,000 K generated via a machine-learning potential and first-principles calculations. We use the Smooth Overlap of Atomic Positions descriptor (SOAP), principal component analysis (PCA), and k-nearest-neighbor distances in the full descriptor space. PCA shows that the dominant variation largely follows density-driven packing, whereas full-space distances reveal pronounced differences in structural similarity among MgSiO_3_ polymorphs and the liquid. Pyroxene-family phases, majorite, and akimotoite remain comparatively close to the liquid, but bridgmanite and postperovskite are systematically less liquid-like. This separation is not explained by the SiO_4_-to-SiO_6_ coordination change alone, and instead stems from a coupled variation in multiple structural features. These results show that local atomic descriptors expose structural distinctions hidden by conventional averaged metrics and suggest that high-pressure liquid thermodynamics, including the nonmonotonic pressure dependence of liquid Grüneisen parameters, need not be interpreted simply as progressive convergence toward the stable lower-mantle crystal structures.

Significance Statement The atomic structures of silicates play a fundamental role in determining their thermophysical properties. Structural relationships among different phases are traditionally interpreted using aggregate structural observables, which do not directly quantify the similarity of individual atomic environments across multiple phases. Using Smooth Overlap of Atomic Positions, a local atomic descriptor, we compare major MgSiO_3_ polymorphs and the liquid over a broad pressure–temperature range. We show that structural similarity is not controlled by the well-known transition from tetrahedral to octahedral silicon coordination alone, but by several structural features. Thus, dense silicate liquids can remain structurally distinct from stable lower-mantle crystal structures even as they acquire increasingly solid-like thermodynamic behavior. Our work establishes a quantitative framework for comparing local structure across complex phase diagrams.

## Introduction

MgSiO_3_ is a major constituent of Earth’s mantle and likely of many rocky exoplanets (1, 2). Its crystalline and liquid phases play fundamental roles in magma-ocean crystallization (3), lower-mantle mineralogy, mantle differentiation, and the evolution of terrestrial planets (4, 5). Structural transitions in MgSiO_3_, particularly the emergence of bridgmanite and postperovskite at high pressure, strongly affect density (6), seismic properties, elasticity (7, 8), thermal and electrical transport (9, 10), and mantle dynamics (11). In silicate liquids, changes in local atomic environments with pressure and temperature are closely linked to viscosity, diffusivity, and crystallization pathways (12). Understanding the structure of MgSiO_3_ across crystalline and liquid phases is therefore central to mineral physics, geodynamics, and planetary science. Yet despite decades of research on MgSiO_3_ polymorphs and liquids, it remains unclear how local atomic environments are related across the diverse crystalline and liquid phases that populate its phase diagram.

The structure of silicates has traditionally been characterized using diffraction- and simulation-derived observables such as structure factors, radial distribution functions, bond-angle distributions, and coordination-number statistics. These approaches have provided fundamental insights into pressure-induced coordination changes, short-range ordering, and the local geometry of crystalline and amorphous silicates. In MgSiO_3_, analyses based on Si–O coordination, pair correlations, and bond-angle distributions have played a central role in understanding structural evolution across polymorphs and liquid phases (12–16).

Despite their importance, these descriptors do not provide a direct quantitative measure of local-environment similarity across multiple crystalline and liquid phases. Coordination numbers, radial distribution functions, and bond-angle distributions are aggregate observables that characterize specific aspects of structure but do not directly compare individual atomic environments across phases. As a result, it remains difficult to quantify which local motifs are shared among polymorphs and the liquid, which structures are fundamentally distinct, and what structural features dominate phase-to-phase similarity in complex silicates such as MgSiO_3_.

Local atomic descriptors provide a new framework for comparing local structure across chemically and structurally diverse phases. In particular, the Smooth Overlap of Atomic Positions (SOAP) descriptor represents local atomic neighborhoods in a rotationally, translationally, and permutationally invariant high-dimensional space, enabling direct quantitative comparison between individual atomic environments (17). SOAP and related local atomic descriptors have been widely used in machine-learning potentials (18–21) and, more recently, to investigate structural relationships among phases. For example, descriptor-space analyses have shown that liquid water contains local environments resembling those found in multiple crystalline ice polymorphs (22). These approaches offer the possibility of comparing crystalline polymorphs and liquid within a common descriptor space beyond conventional low-order structural metrics.

Here, we analyze the local structural similarity among major MgSiO_3_ polymorphs and liquid across 0.5–700 GPa and 500–12,000 K using SOAP descriptors, principal component analysis (PCA), and nearest-neighbor similarity metrics (Figs. 1 and S1). We compare local environments in enstatite, majorite, akimotoite, bridgmanite, postperovskite, and liquid MgSiO_3_ to determine which structural motifs are shared across phase space and what structural features control their similarity. While low-dimensional projections suggest broad overlap among many phases, analysis in the full descriptor space reveals that bridgmanite and postperovskite remain the most structurally distinct phases. We further show that this separation is not governed solely by the SiO_4_-to-SiO_6_ coordination transition (15, 23), but instead by a variety of structural changes to the species-specific local environments. These results establish a quantitative framework for comparing local structure across silicate phase space and demonstrate how local atomic descriptors can reveal structural relationships beyond conventional coordination-based analysis.

**Figure 1:**
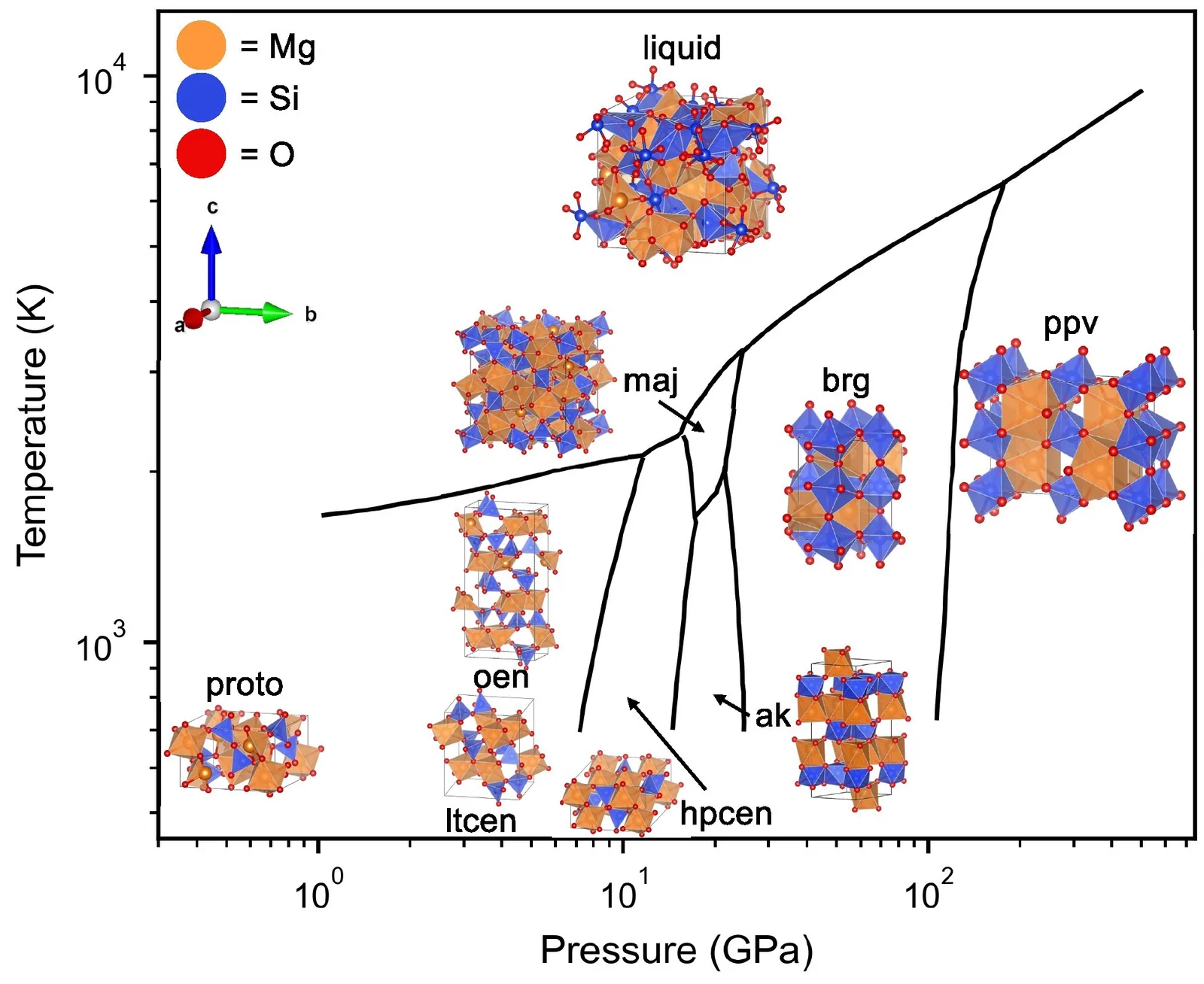
Phase diagram with several liquid and crystalline structures of MgSiO_3_: akimotoite (ak), bridgmanite (brg), high-pressure clinoenstatite (hpcen), low-temperature clinopyroxene (ltcpx), majorite (maj), orthoenstatite (oen), postperovskite (ppv), and protoenstatite (proto). The solid–solid, enstatite-liquid, and majorite-liquid phase boundaries are constructed based on the experimental results gathered by Duffy et al. (24) and the bridgmanite and postperovskite liquidus curves come from Deng et al. (25).

## Methods

### Simulations and data post-processing

Molecular dynamics simulations were conducted using the Large-scale Atomic/Molecular Massively Parallel Simulator (LAMMPS) (26) with DeepMD-Kit (27, 28), and the machine-learning potential developed by Peng and Deng (9) which was trained on configurations of pure and hydrous bridgmanite, postperovskite, and MgSiO_3_ liquid. We also tested this potential on the lower-pressure, lower-temperature phases of MgSiO_3_ not included in the training dataset, and for postperovskite at higher pressure and temperature, and found that it accurately simulates these structures despite these data not being found in the training dataset, probably due to the enhanced sampling approaches adopted in the training process (25). The simulations employed the NPT ensemble with the aniso setting which allows all unit cell lengths to adjust to optimize the unit cell size and shape at a given pressure and temperature. Each run was held at the target temperature and pressure for 100 ps and sampled every 500 fs. The simulation cells range from 80 to 270 atoms depending on the phase.

High-pressure, high-temperature liquid runs outside of the training distribution of the machine-learning potential were generated via ab initio molecular dynamics (AIMD) with the Vienna Ab Initio Simulation Package (VASP) (29) version 6.3.4 with the projector augmented wave method (30) and the PBEsol approximation (31). The projector augmented wave method (PAW) potentials contained the following valence electrons: Mg $2p^{6}3s^{2}$, Si $3s^{2}3p^{2}$, and O $2s^{2}2p^{4}$. An energy cutoff of 800 eV, timestep of 1.0 fs, and *Γ*-point Brillouin zone sampling were selected, following the settings of Luo and Deng (12) for silicate melts. Each 160-atom liquid was simulated in the NVT ensemble for several ps with the initial transient portion of each trajectory excluded from the structural analysis after the energy had stabilized. Cell parameters were adjusted before production runs to reduce deviatoric components of the time-averaged stress.

In total, 134 independent runs were performed across the nine phases (Fig. S1), yielding ∼63,600 atomic configurations. Simulations in this study were conducted from 0.5 to 700 GPa and from 500 to 12,000 K.

### SOAP descriptors for local atomic environments

The SOAP descriptor (17) represents the local atomic environment by constructing a smooth neighbor density from Gaussian functions centered on neighboring atoms within a spherical cutoff radius. This density is subsequently expanded in radial basis functions and spherical harmonics to encode information about the distances, angles, and chemical species of nearby atoms.

First, for each central atom *i*, a smooth neighbor density is constructed by placing Gaussian functions at the positions of neighboring atoms *j*:


(1) $$\rho_{i}(r)=\sum_{j}\exp\;\left(-\frac{{\left|r-r_{ij}\right|}^{2}}{2\sigma^{2}}\right),$$

where *σ* controls the width of the Gaussian functions. For systems containing multiple chemical species, separate neighbor-density channels distinguish different atomic species.

Next, a basis expansion projects the density $\rho_{i}$ onto radial basis functions $g_{n}(r)$ and spherical harmonics $Y_{lm}(\hat{r})$:


(2) $$\rho_{i}(r)=\sum_{nlm}c_{nlm}^{\left(i\right)}g_{n}(r)Y_{lm}(\hat{r}),$$

where $c_{nlm}^{\left(i\right)}$ is the expansion coefficient for the local environment of atom *i*. The radial and angular resolution of the representation can be controlled through the number of radial basis functions and maximum angular degree included in the expansion. Finally, rotationally invariant features are obtained by constructing the power spectrum *p* and summing over the magnetic quantum number *m*:


(3) $$p_{nn^{\prime }l}^{\left(i\right)}=\sum_{m}c_{nlm}^{\left(i\right)}c_{n^{\prime }lm}^{(i)*}.$$

The resulting SOAP vector encodes rotationally invariant radial and angular correlations within a cutoff radius of the atom of interest.

The SOAP vectors were generated with the DScribe python package (32), using a cutoff radius ($r_{\mathrm{cut}}$) = 6 Å, number of radial basis functions ($n_{\max}$) = 6, maximum degrees of spherical harmonics ($l_{\max}$) = 6, and Gaussian smearing width (*σ*) = 0.44 Å. 6,834 configurations were selected for SOAP analysis, comprising ∼1.09 million Mg-, Si-, and O-centered local atomic environments. SOAP descriptors were computed in parallel on four cores with 32 GB RAM.

### Principal component analysis

Principal component analysis (PCA) (33, 34) was used to visualize the dominant modes of variation in the SOAP descriptor space. SOAP vectors are high-dimensional vectors that can be difficult to compare without dimensionality reduction. For example, using the parameters we selected for SOAP, there are 1,197 features in the SOAP vector. PCA conveniently reduces the number of features to those that explain the most variance, allowing for more interpretable visualization of the data.

For each atomic species, SOAP vectors were projected onto the eigenvectors of their covariance matrix, with the corresponding eigenvalues giving the variance explained by each principal component. The resulting PCA coordinates provide a low-dimensional representation of the largest structural variations among local atomic environments. PCA was calculated separately for each species; therefore, the principal components do not necessarily capture the same information in each separate PCA analysis. Because dimensionality reduction can obscure distances in the original descriptor space, PCA was used for visualization and interpretation, whereas quantitative liquid-crystal similarity was evaluated using k-nearest-neighbor (kNN) distances in the full SOAP space (K-nearest neighbors distance section).

PCA was conducted on the SOAP transformed local atomic environments with 10 principal components selected. No additional normalization or standardization was applied prior to PCA so that the principal components reflected the dominant variations in the original SOAP representation. These two techniques would alter the magnitude and variance of the SOAP vectors, two properties that could contain useful information about the similarities and differences between phases. Therefore, using the unscaled vectors allowed PCA to discover the directions of greatest variation in the SOAP feature space without imposing any feature-scaling transformations.

Environments were sampled for principal component analysis in order to maintain equal amounts of environments for each phase, so as not to bias the model toward variation in any one phase. For example, only one simulation of protoenstatite was conducted due to its relatively small stability region, while there were 78 runs just for liquid. For fairness, half of the equilibrated frames from the protoenstatite run were used and an equal number were evenly sampled across all of the liquid simulations and PCA was computed using these sampled environments. Then, every atom from the full SOAP dataset was projected back onto these principal components for final analysis and visualization (Fig. 2). PCA was computed on a single NVIDIA A100 using the cuML library (35).

**Figure 2:**
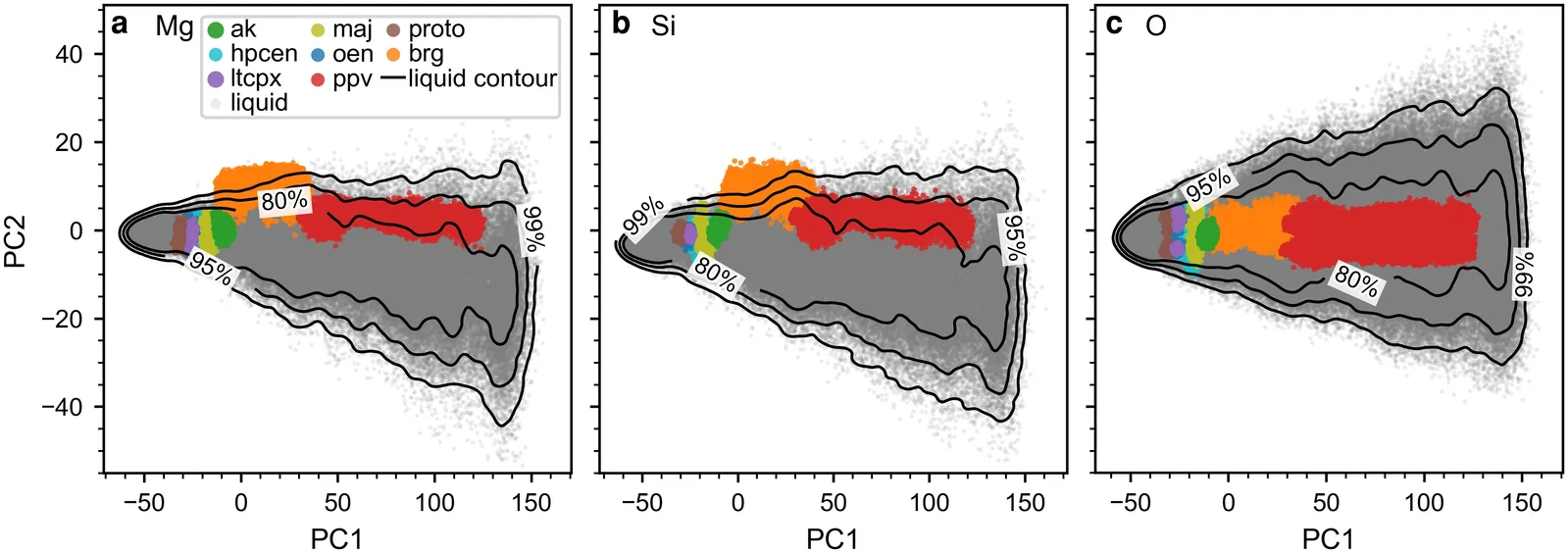
PCA of SOAP descriptors for a) Mg-, b) Si-, and c) O-centered local atomic environments in MgSiO_3_ polymorphs and liquid. Contours enclosing 80, 95, and 99% of liquid environments are superimposed to illustrate the structural diversity of the liquid in the projected descriptor space. Many crystalline environments fall within the liquid contours, indicating substantial overlap in the dominant structural variance captured by the first two principal components. Liquid density contours were obtained by binning the liquid PC1–PC2 distribution into a 2D histogram, smoothing with a Gaussian filter, and selecting density thresholds enclosing 80, 95, or 99% of total smoothed liquid density.

Density, pressure, and temperature were calculated by averaging the instantaneous values across the equilibrated frames from each MD trajectory and plotted as heatmaps over the principal components (Figs. 3, S2, and S3). Selected descriptors were also calculated from these equilibrated frames and compared to the principal components (Fig. 4).

**Figure 3:**
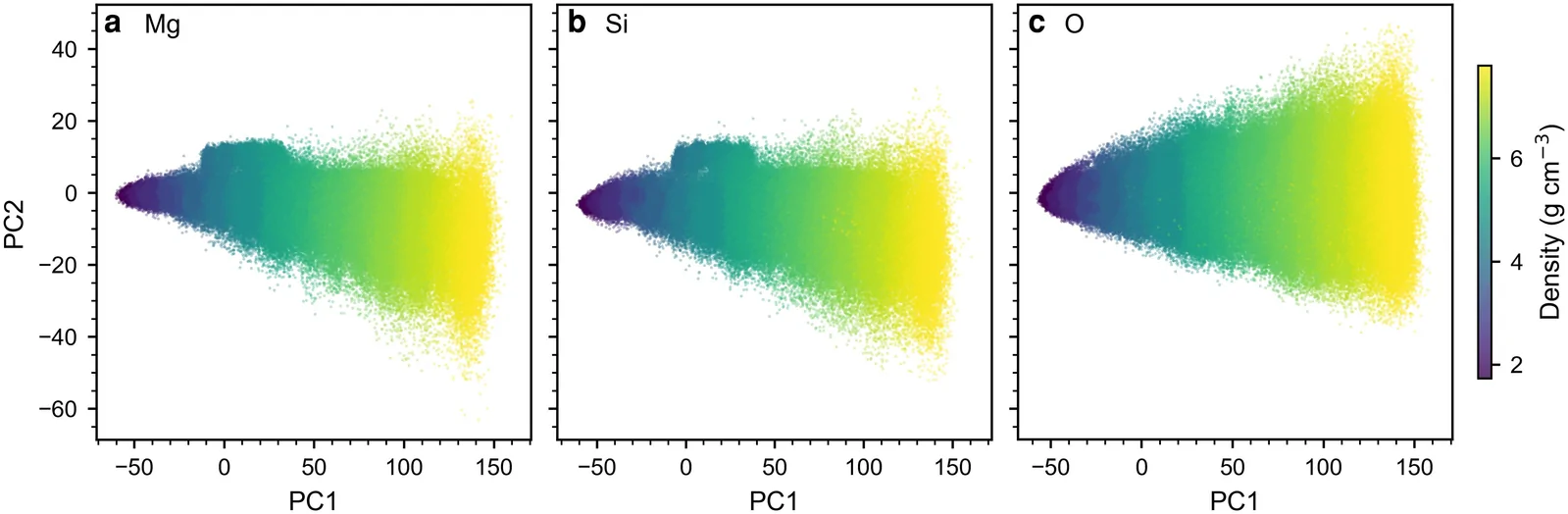
PCA projections of SOAP descriptors for a) Mg-, b) Si-, and c) O-centered local atomic environments in MgSiO_3_ polymorphs and liquid, colored by density. The strong correlation between PC1 and density indicates that compression-induced densification accounts for much of the structural variance captured by the leading principal component.

**Figure 4:**
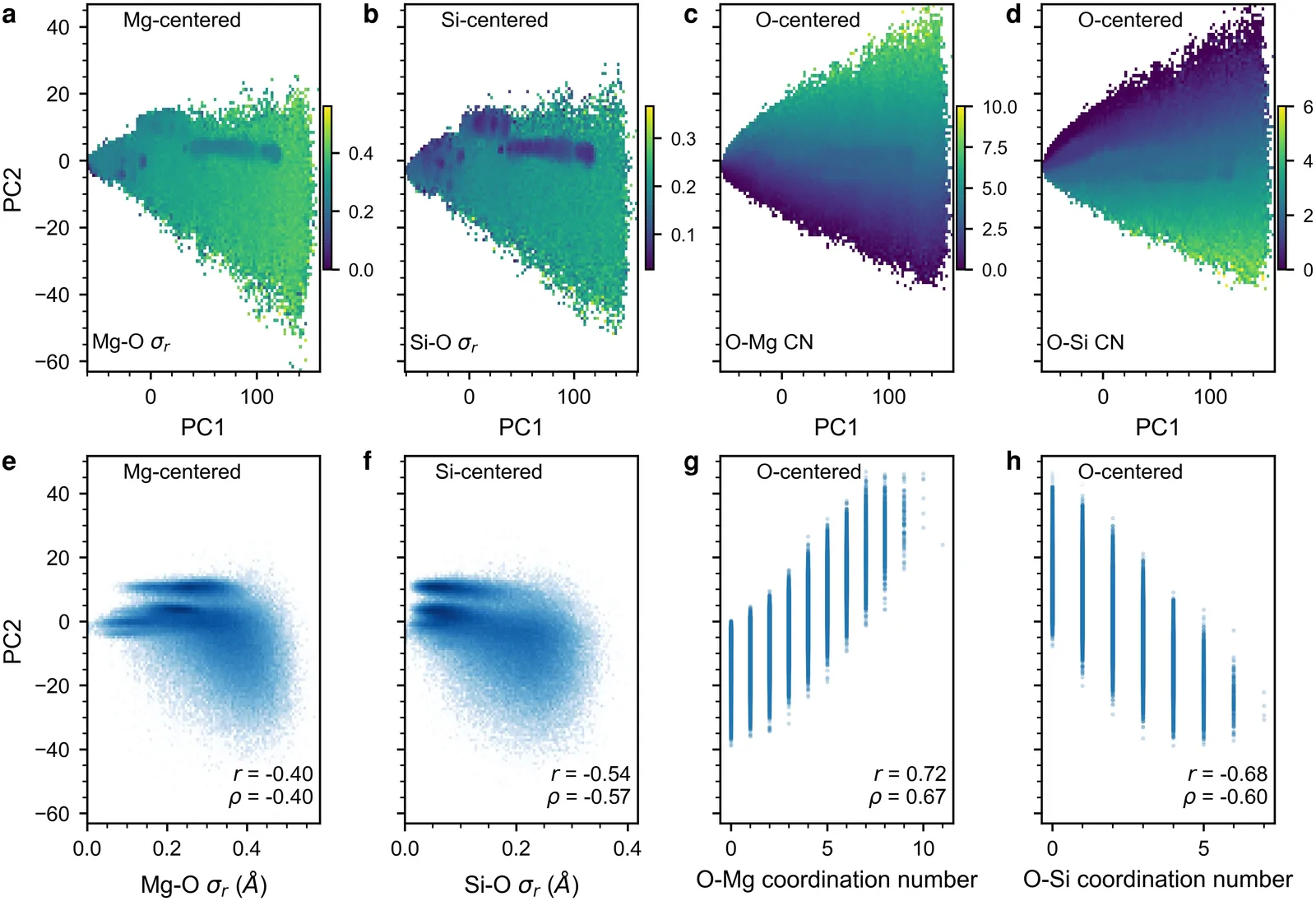
Selected structural metrics show that PC2 reflects different changes in local structure depending on the species. The top row plots PC1–PC2 projections of atom-centered SOAP environments colored by the most strongly correlated features: a) Mg–O bond length SD ($\sigma_{r}$) for Mg-centered environments, b) Si–O bond length SD for Si-centered environments, c) O–Mg CN for O-centered environments, and d) O–Si CN for O-centered environments. The bottom row plots the same metrics directly against PC2, with Pearson (r) and Spearman (*ρ*) reported in each panel. PC2 shows moderate negative correlations with Mg–O $\sigma_{r}$ and Si–O $\sigma_{r}$, and stronger correlations with O–Mg (positive) and O–Si (negative) coordination.

### K-nearest neighbors distance

kNN distances (36) were used to quantify local structural similarity between SOAP environments. For each central atom type separately (Mg, Si, and O), SOAP vectors from a query phase were compared with SOAP vectors from a reference phase. For each query environment, Euclidean distances were calculated to environments of the same central atom type in the reference phase. Then, these distances were sorted and the k lowest distances were selected to represent the kNN distances. The mean distance to these k-nearest reference neighbor environments was used as the local kNN distance for that query environment. Phase-pair distances were then computed by averaging these local distances over all environments in the query phase. A value of k=10 was used for this study.

This procedure was repeated for all pairs of phases, including liquid, yielding phase-pair distance matrices for Mg-, Si-, and O-centered environments. Smaller mean 10-nearest neighbor distance values indicate that local environments in the query phase are, on average, more similar to those within the reference phase, whereas larger values indicate more dissimilar local environments in SOAP space. These phase-averaged distances are reported in Fig. 5. For example, for query phase bridgmanite and reference phase liquid, the mean distance of the average bridgmanite Mg-centered environment to its 10-nearest liquid Mg-centered environments is low (7.3). However, when the query phase is liquid and the reference phase is bridgmanite, the mean distance of the average Mg-centered liquid environment to its 10-nearest bridgmanite neighbors is larger (39.4). This is because the bridgmanite Mg-centered environments are relatively clustered in SOAP space while the liquid Mg-centered environments cover a much larger range of SOAP space. Therefore, there will be many liquid environments that are very far from bridgmanite environments in SOAP space, which will drive up the average distance. The scikit-learn NearestNeighbors implementation was used to efficiently calculate pairwise distances (37).

**Figure 5:**
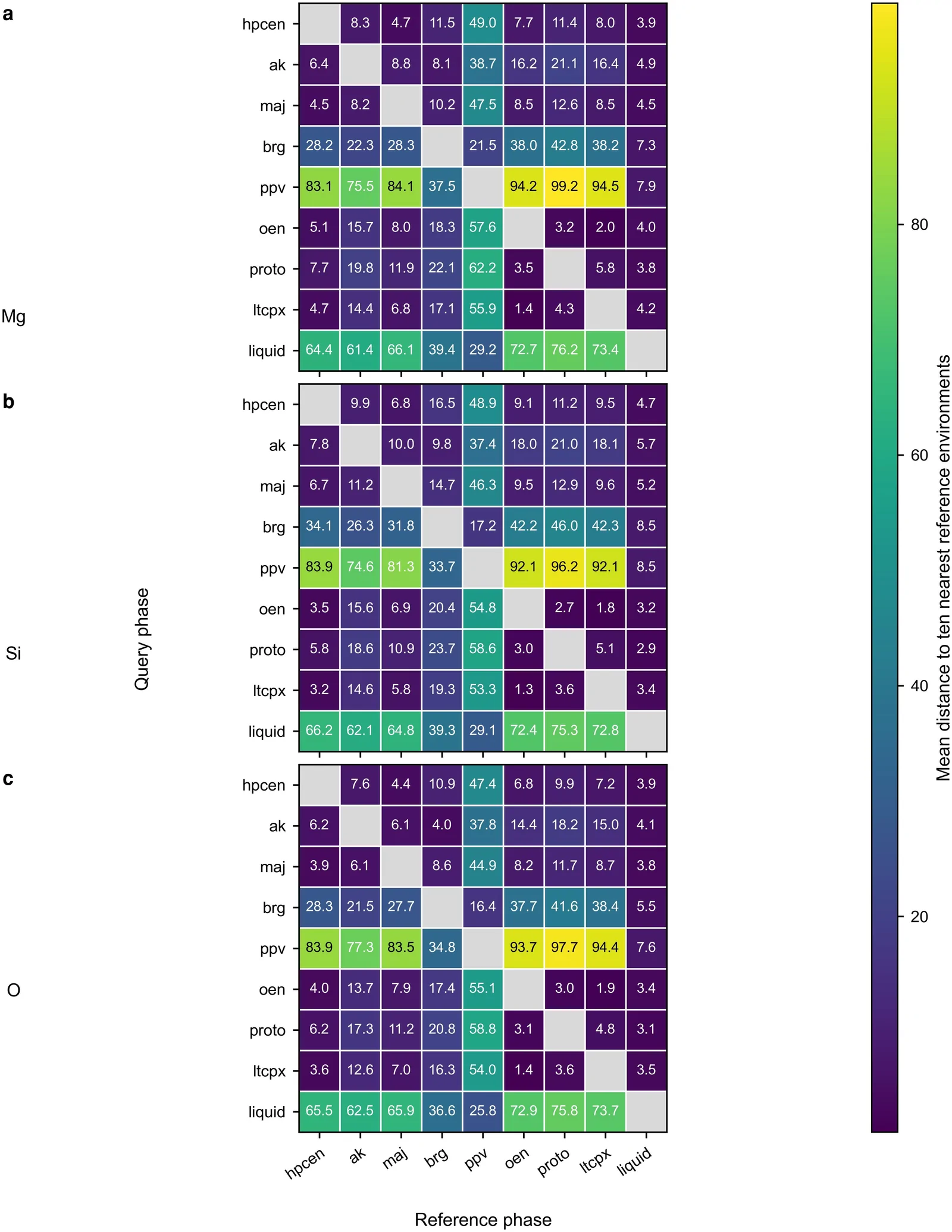
Heatmap of the mean Euclidean distance between the SOAP vector of each atomic environment in the query phase (rows) to the SOAP vectors of its 10 nearest neighbors in the reference phase (columns) for (a) Mg, (b) Si, and (c) O. Postperovskite and bridgmanite are notably further from most of the low-pressure phases. Phases are akimotoite (ak), bridgmanite (brg), high-pressure clinoenstatite (hpcen), low-temperature clinopyroxene (ltcpx), majorite (maj), orthoenstatite (oen), postperovskite (ppv), and protoenstatite (proto).

To examine the crystal-liquid comparison in more detail, the distributions of individual kNN distances from each crystalline phase to liquid are shown in Fig. 6.

**Figure 6:**
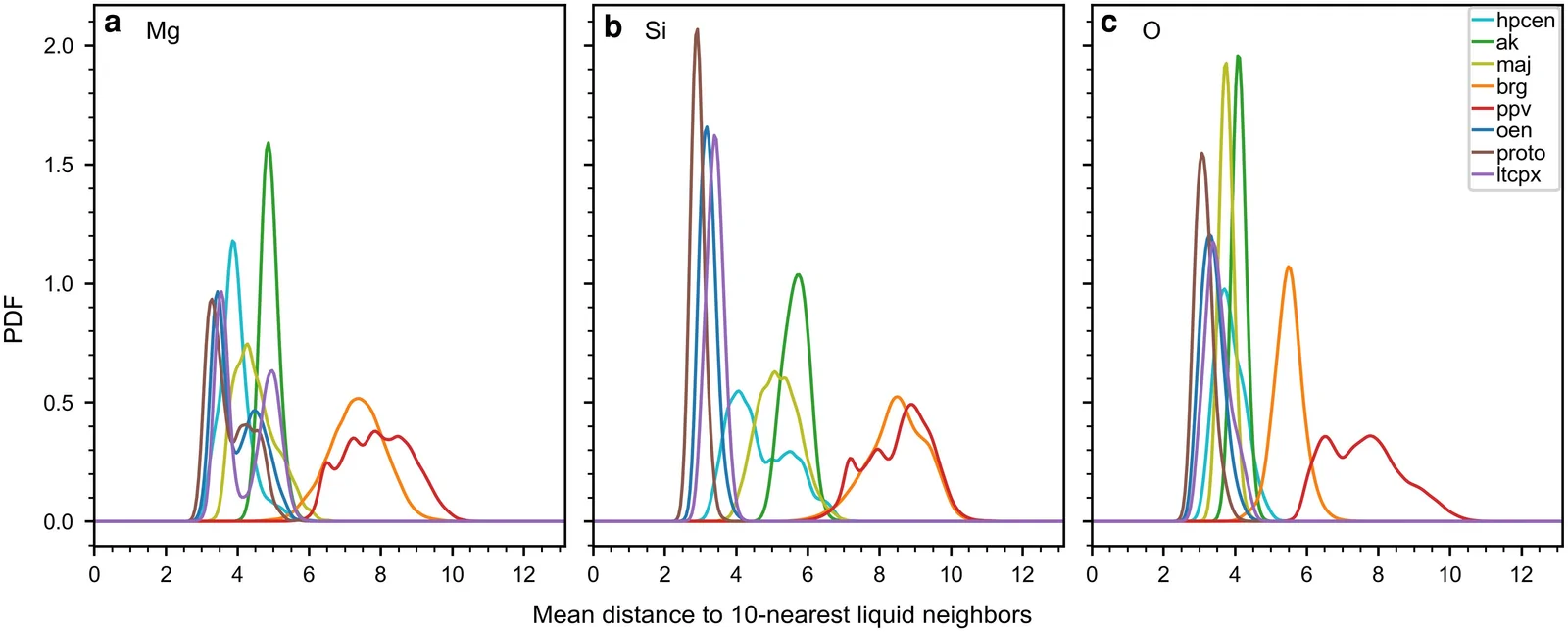
Distributions of the mean distances from the SOAP vectors of each crystalline phase to the SOAP vectors of its 10 nearest liquid environments for (a) Mg, (b) Si, and (c) O. For each local atomic environment of a given element in each crystalline phase, the unitless Euclidean distances between its SOAP vector and the SOAP vectors of all of the local atomic environments of the same element in the liquid are calculated. Then, the mean of the 10 closest of these distances is computed. Phases are: akimotoite (ak), bridgmanite (brg), high-pressure clinoenstatite (hpcen), low-temperature clinopyroxene (ltcpx), majorite (maj), orthoenstatite (oen), postperovskite (ppv), and protoenstatite (proto).

## Results and discussion

We used PCA to identify the dominant sources of variation in the SOAP descriptors of Mg-, Si-, and O-centered local atomic environments across eight crystalline phases of MgSiO_3_ and the liquid. The first two principal components (PC1 and PC2) were projected to visualize structural relationships among phases in a low-dimensional representation of the descriptor space (Fig. 2). In the resulting PCA projections, local environments from each phase occupy distinct but partially overlapping regions. The relative positions of these regions provide a qualitative view of structural relationships among phases, although overlap in low-dimensional PCA space does not necessarily imply similarity in the full SOAP descriptor space. In PCA space, the local atomic environments of Mg and Si in the crystalline phases mostly overlap with those in the liquid with some exceptions (Fig. 2). In contrast, the local atomic environments of O in crystals overlap entirely with those in the liquid. This demonstrates overlap in the leading PCA dimensions between crystalline phases and the liquid, although it does not establish similarity in the full SOAP space. Nevertheless, bridgmanite exhibits systematic deviations from the liquid distribution in both Mg- and Si-centered environments, suggesting structural features that are less well represented in the liquid. Specifically, the bridgmanite clusters are displaced toward larger PC2 values relative to the liquid trend, and portions of the Mg and Si distributions extend beyond the PC2 range sampled by the liquid at comparable PC1 values. In contrast, postperovskite extends along PC1 but remains close to the central PC2 axis of the liquid, indicating that its distinctiveness is not captured by the leading principal components. The displacement of bridgmanite along PC2 is consistent with its highly coordinated Mg A-site environment paired with sparse Mg–Mg neighbors (38), a structural motif that appears to be less frequently sampled in the liquid at comparable density.

We quantified phase overlap with the liquid using the contours in Fig. 2 (Table S1; contour calculation in caption). In general, most phases fall within all three contours. Bridgmanite is a clear exception: only 2.5% of its Mg environments and 3.3% of its Si environments fall within the 80% liquid contours, and even the 99% liquid contours capture only 55.2% (Mg) and 55.6% (Si) of bridgmanite environments. In contrast, postperovskite Mg and Si environments lie almost entirely within the 99% liquid contour (100%), and nearly all O environments of the crystalline phases are fully contained within the 99% liquid contour. Overall, the contours demonstrate that Mg- and Si-centered phase separation is apparent in the PC1–PC2 projection, but higher dimensional analysis is needed to separate the phases using the O-centered environments.

Coloring the PCA projections by density reveals a strong correlation between PC1 and density for Mg-, Si-, and O-centered environments, with higher PC1 values corresponding systematically to higher densities (Fig. 3a). Both crystalline and liquid phases exhibit a continuous density trend along PC1, extending from low-pressure polymorphs such as protoenstatite to high-pressure phases such as postperovskite. The liquid spans nearly the entire density range sampled by the crystalline polymorphs and extends to densities exceeding those represented in the available crystal dataset.

Coloring the PC1 vs. PC2 plot by pressure reveals a similar trend to that of density, because pressure and density are positively correlated (Fig. S2). However, this relationship between PC1 and pressure is not as uniform as for density because for a given density, the crystal occurs at a lower pressure than the equally dense liquid. Similarly, temperature is also roughly correlated with PC1 because the temperature and pressure of these simulations are correlated (Fig. S3). As with pressure, the temperature of a crystal at a given density is lower than that of an equally dense liquid.

To interpret the structural meaning of PC2, we compared the PCA projections with several local geometric descriptors and found that PC2 correlates weakly for several descriptors for Mg- and Si-centered environments, and correlates strongly with first-shell coordination numbers for O-centered environments (Fig. 4). The distances used to approximate the first shell of atoms were taken from the first troughs in the radial distribution functions (RDFs) of the different bonds in liquid MgSiO_3_ determined by Deng and Stixrude (39). A single value for the trough was selected because these bond lengths are found to be fairly consistent across pressure and density changes in the liquid at high pressures (12). While using a single cutoff radius across different crystals is not standard practice, this choice was made for computational simplicity and has little effect on the analysis. For all phases except bridgmanite, the ranges of PC2 values spanned by the local atomic environments in the crystal are within the range of values found in the liquid.

For Mg-centered environments, PC2 is negatively correlated with Mg–O bond-length variability and the number of neighbors within 6 Å, and weakly positively correlated with mean Mg–O bond length. For Si-centered environments, PC2 is negatively correlated with Si–O bond-length variability and neighbor counts, and positively correlated with the mean O–Si–O angle. O–Si–O angular variability shows a positive Spearman correlation but only a weak Pearson correlation (Table S2). Bond length and angle SD are measures of polyhedral distortion. The observed association with distortion may contribute to the PC2 separation.

For O-centered environments, PC2 has strong positive correlation with the O–Mg CN and strong negative correlation with the O–Si CN. The strongest correlated features for PC2 were plotted (Fig. 4). This inverse relationship between the O–Mg CN and the O–Si CN is consistent with the charge balance relationship between Mg and Si, where O-centered environments with higher O–Mg coordination tend to have lower O–Si coordination in the sampled dataset.

The moderate correlations of geometric descriptors to PC2 support the usage of atomic descriptors such as SOAP to evaluate crystal and liquid similarity. Because no single descriptor correlates perfectly with any principal component, especially for PC2 in Mg- and Si-centered environments, this demonstrates how SOAP combines multiple types of structural information (radial and angular correlations up to three-body order) to build a more complete description of the local atomic structure than a simple descriptor such as a coordination number. Therefore, SOAP may find distinctions between phases where simple descriptors alone may fail to separate them.

The explained variance ratio of the principal components was >95% for PC1 in all three species (Mg, Si, O), 1.0–1.9% for PC2, and <1% for each of the remaining principal components. This demonstrates that bulk density in the 6 Å window is by far the dominant source of variation in local structure for all three species, while various structural components provide a secondary and largely independent axis.

### K-nearest neighbors distances

To quantify structural similarity beyond the low-dimensional PCA projections, we calculated kNN distances between local environments in all crystalline phases and the liquid (Fig. 5). The kNN metric measures the average SOAP-space distance between local environments and their 10 nearest neighbors in another phase, providing a quantitative measure of phase-to-phase structural similarity. Smaller distances indicate greater similarity, whereas larger distances indicate more distinct local environments.

The low-pressure phases exhibit low mean distances, indicating similarities in local structure between local atomic environments in these species. In contrast to the substantial overlap observed in PCA space, bridgmanite and postperovskite exhibit systematically larger kNN distances to both the liquid and the lower-pressure polymorphs (Fig. 5). These two phases form a distinct structural group despite sharing the same MgSiO_3_ composition and broadly similar coordination states. Because density and first-shell coordination are already largely represented by PC1 and PC2, the additional separation revealed by the kNN analysis must arise from structural information encoded in the full SOAP descriptor space, which is correlated with bond angles, oxygen packing, and polyhedral distortion.

The polymorphs of MgSiO_3_ span a wide range of polyhedral connectivities, Si coordination states, and polyhedral arrangements across the mantle pressure range (Fig. 1). The low-pressure pyroxene-family phases contain corner-sharing chains of SiO_4_ tetrahedra, whereas majorite adopts a garnet structure in which most Si remains tetrahedrally coordinated while a minority occupies octahedral sites. Akimotoite is the lowest-pressure MgSiO_3_ polymorph in which all Si is octahedrally coordinated, forming layered networks of SiO_6_ and MgO_6_ octahedra connected through edge- and face-sharing. Bridgmanite adopts the perovskite structure, where corner-sharing SiO_6_ octahedra form a 3D network surrounding highly coordinated Mg A-site cages. Postperovskite consists of layered SiO_6_ octahedral sheets in which octahedra share both edges and corners, producing a topology distinct from the 3D framework of bridgmanite (6, 7). Thus, although akimotoite, bridgmanite, and postperovskite all contain fully octahedral Si, they realize the pressure-driven coordination transition through fundamentally different polyhedral arrangements and connectivities.

The distributions of average distances to the 10 nearest liquid environments were analyzed as probability density functions (Fig. 6). The pyroxene-family phases and majorite exhibit narrow distributions centered at low distances, indicating strong local structural similarity to the liquid. Akimotoite, the first fully octahedral SiO_6_ polymorph in the pressure sequence, shows a moderate increase in Si-environment distance (mean 5.7) while remaining comparatively similar to the liquid in Mg and O. Majorite and high-pressure clinoenstatite exhibit slightly broader Si distributions that are slightly offset from the lower pressure phases (means 5.2 and 4.7) indicating slight structural differences between Si environments in the liquid and these two phases.

Bridgmanite and postperovskite are systematically the most distant phases from the liquid in all three species, although the origin of their separation differs. Bridgmanite exhibits comparable shifts in Mg, Si, and O environments (means 7.3, 8.5, and 5.5), whereas postperovskite is distinguished by all three environments (means 7.9, 8.5, and 7.6) but significantly more offset in O, exceeding the bridgmanite O distance by ∼38%. Both lower-mantle polymorphs are structurally distinct from the liquid, displaying a broadly distributed mismatch across species.

These results demonstrate that the breakdown of liquid–crystal similarity is not controlled solely by the pressure-induced transition from SiO_4_ to SiO_6_ coordination. Akimotoite, despite being the first phase in which all Si atoms become octahedrally coordinated, remains substantially closer to the liquid than either bridgmanite or postperovskite. The coordination transition therefore represents only part of the structural evolution across the MgSiO_3_ phase diagram. Additional features encoded in the full SOAP descriptor space—mixtures of radial and angular information—play a dominant role in determining phase-to-phase similarity.

The kNN analysis should be interpreted in the context of the structural heterogeneity of MgSiO_3_ liquid (40). Rather than exhibiting a single coordination state, the liquid contains a broad distribution of SiO_n_, MgO_n_, and O-centered environments. Recent ab initio molecular dynamics (AIMD) simulations extending to terapascal pressures have shown that the relative abundance of these environments evolves continuously with pressure and temperature (12). In this context, a small nearest-neighbor distance to the liquid does not imply global structural equivalence between a crystal and the liquid. Instead, it indicates that the liquid ensemble contains local atomic environments closely resembling those found in the crystalline phase.

The kNN analysis therefore moves beyond coordination statistics by asking whether the complete SOAP environment, including local geometry, neighbor arrangement, and higher-order structural organization, is represented within the liquid ensemble. This distinction is particularly important because akimotoite, bridgmanite, and postperovskite all contain fully octahedrally coordinated Si yet exhibit markedly different distances from the liquid. The larger distances of bridgmanite and postperovskite therefore cannot be attributed simply to the presence of SiO_6_ units. Instead, they reflect differences in Mg-, Si-, and O-centered environments that are not captured by coordination number alone.

## Implications

Our results suggest a broader role for local atomic descriptors in mineral physics, complementary to conventional structural observables. Rather than replacing conventional structural observables, local-environment descriptors provide complementary perspectives by quantifying structural relationships across complex phase diagrams. For example, even when environments in the liquid and crystal polymorphs share the same coordination number, they may still have significant differences in their SOAP vectors. The same framework should be readily applicable to other silicates, oxides, and planetary materials where coordination changes alone do not fully capture structural complexity.

More importantly, using a local atomic descriptor provides a structural language that applies to both crystals and liquid. Crystal descriptors often rely on coordination polyhedra, symmetry, and connectivity, whereas liquids are described by coordination distributions and RDFs. SOAP, and other local atomic descriptors, unify the analysis using a single mathematical representation. In addition, the structural trends identified here may also have implications for understanding the thermodynamics of compressed silicate liquids.

A widely invoked qualitative picture is that nonmetallic liquids become progressively more “solid-like” under compression and therefore gradually acquire thermodynamic behavior resembling that of crystalline solids (41, 42). This interpretation is often used to rationalize the pressure dependence of the Grüneisen parameter in silicate liquids (and liquid Fe (43)), which commonly increases at low pressures before eventually plateauing or decreasing with compression, approaching the monotonic behavior observed in solids as reported in previous theoretical and experimental studies (12, 44, 45).

Our results suggest that this interpretation is incomplete for MgSiO_3_. Although compression produces substantial densification and drives the liquid toward higher coordination states, the liquid does not become uniformly closer to the stable lower-mantle crystal structures in descriptor space. Bridgmanite and postperovskite remain the most structurally distinct phases, whereas akimotoite remains comparatively close to the liquid despite sharing the same SiO_6_ coordination state. The relevant distinction is therefore not simply coordination or density, but the emergence of specific differences in species-dependent local atomic environments that are not fully represented in the liquid.

If structural similarity to crystalline phases in descriptor space serves as a proxy for thermodynamic similarity, then viewed in this way, the evolution of the liquid Grüneisen parameter need not be interpreted as progressive convergence toward the stable lower-mantle crystal structures. Instead, it may reflect a crossover within the liquid itself: from a relatively open liquid to a densely packed, thermodynamically stiffer liquid whose local atomic environments remain distinct from bridgmanite- and postperovskite-like frameworks. In this picture, thermodynamic “solid-likeness” and structural similarity to the equilibrium crystal are not equivalent concepts. A liquid may acquire increasingly solid-like thermodynamic behavior while remaining structurally separated from the stable crystalline polymorphs in local-environment space.

Future work combining pressure-resolved descriptor analysis with calculations of the liquid Grüneisen parameter would provide a direct test of whether the thermodynamic crossover observed in compressed silicate liquids correlates with changes in local structural diversity, coordination saturation, or proximity to specific crystalline topologies. If confirmed, such a relationship would establish a direct connection between local atomic descriptions of local structure and the thermodynamic behavior of planetary materials at extreme conditions.

Local atomic descriptor-space analysis may also provide an atomistic framework for interpreting experimental probes of short-range structure, such as diffraction-derived pair correlations and coordination trends. Because SOAP retains information beyond pairwise radial structure, comparison between these observables and changes in local-environment distributions in SOAP space may help identify the structural origins of experimentally observed trends. Direct quantitative mapping between these observables and SOAP-space similarity remains a topic for future work.

## Conclusion

Conventional structural analyses have provided fundamental insights into silicate structure, but they do not directly quantify the similarity of individual atomic environments across multiple phases. In this study, we applied local atomic descriptors of local atomic environments to compare the structures of major MgSiO_3_ polymorphs and liquid over a broad pressure–temperature range spanning conditions relevant to planetary interiors.

The SOAP analysis reveals that the dominant structural variation across MgSiO_3_ phase space is associated with density-driven packing, and local structural similarity is governed by more subtle factors than density or coordination number alone. While low-dimensional projections suggest substantial overlap between liquid and crystalline phases, analysis in the full descriptor space demonstrates that bridgmanite and postperovskite remain systematically distinct from the liquid. In contrast, akimotoite remains comparatively similar to the liquid despite sharing the same SiO_6_ coordination state as the lower-mantle polymorphs. These results show that the transition from tetrahedral to octahedral silicon is not the principal factor controlling structural similarity. Instead, density, bond-length and angle distributions, polyhedral distortion, coordination, and other higher-dimensional structural correlations jointly distinguish the phases.

Beyond the specific case of MgSiO_3_, this work demonstrates how local atomic descriptors provide information complementary to conventional structural observables. By placing individual atomic environments from crystals and liquids into a common descriptor space, the approach directly quantifies structural similarity across complex phase diagrams and identifies distinctions that are not readily apparent from structurally averaged metrics alone. The framework developed here should be broadly applicable to silicates, oxides, and other planetary materials containing multiple polymorphs and disordered phases.

Finally, our results suggest that thermodynamic evolution and structural evolution need not proceed in parallel in compressed silicate liquids. Although dense liquids may exhibit increasingly solid-like thermodynamic behavior at high pressure, they do not necessarily become progressively more similar to the stable crystalline lower-mantle polymorphs. This distinction provides a framework for interpreting the pressure dependence of thermodynamic properties such as the Grüneisen parameter and motivates future efforts to establish direct links between local structural similarity, liquid thermodynamics, and crystallization pathways in planetary materials under extreme conditions.

## Supplementary Material

pgag347_Supplementary_Data

## Data Availability

The data underlying this article are available on Zenodo at https://doi.org/10.5281/zenodo.20480682. Code is available at https://github.com/jakeacquadro/MgSiO3_Structural_Analysis.
