# Supplementary material for: Local atomic environments reveal structural controls on MgSiO3 liquid-crystal similarity
Source: PNAS Nexus. 2026 Oct 6;5(11):pgag347. doi: 10.1093/pnasnexus/pgag347 (PMC13756074; doi:10.1093/pnasnexus/pgag347)
Supplement: Supplementary file 1 [file pgag347_supplementary_data.pdf]

Supplementary Figures

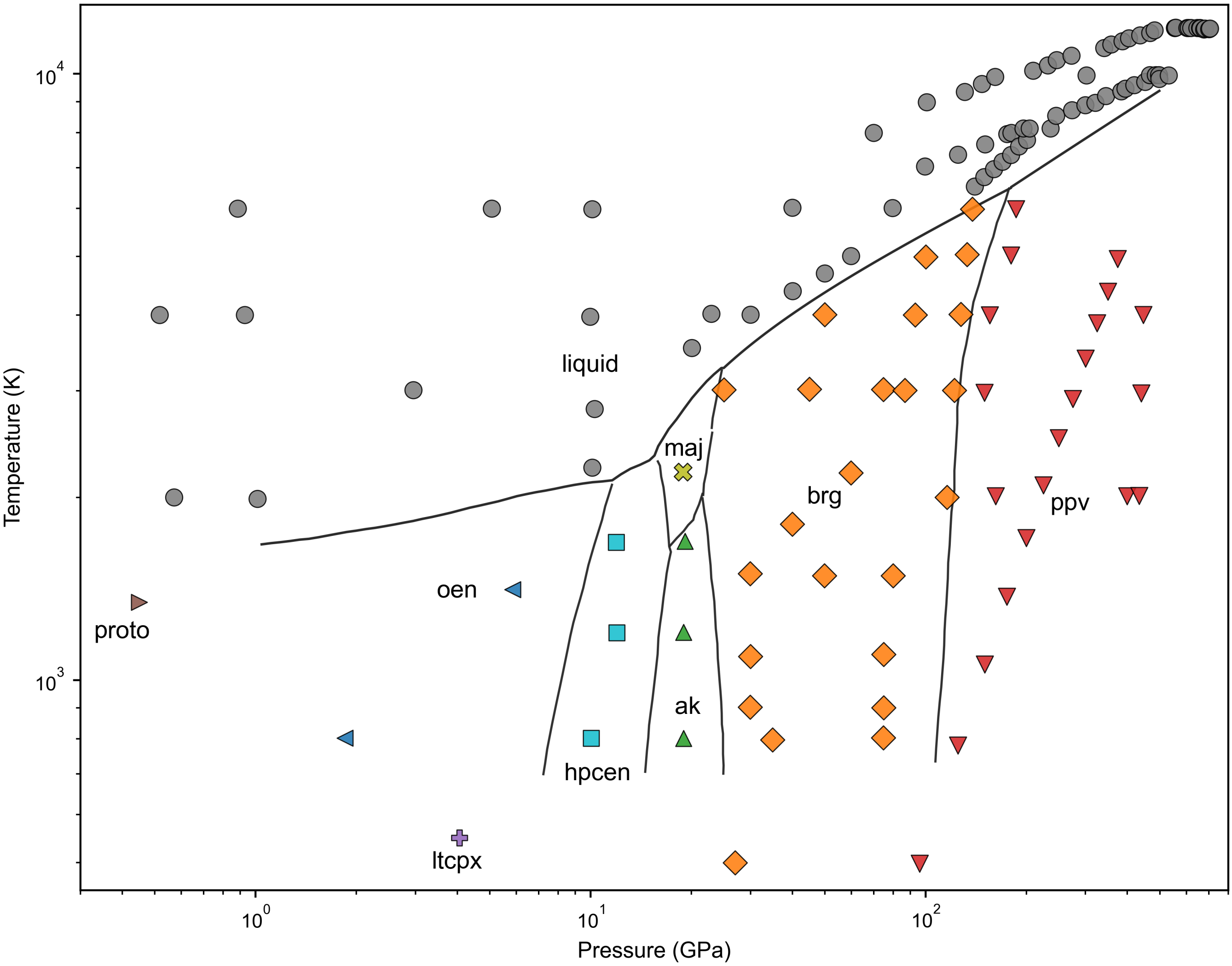

Figure S1. Phase diagram with all liquid and crystalline simulations generated for this study: akimotoite (ak), bridgmanite (brg), high-pressure clinoenstatite (hpcen), low-temperature clinopyroxene (ltcpx), majorite (maj), orthoenstatite (oen), post-perovskite (ppv), and protoenstatite (proto). The solid-solid, enstatite-liquid, and majorite-liquid phase boundaries are constructed based on the experimental results gathered by Duffy et al. (2015) [1] and the bridgmanite and post-perovskite liquidus curves come from Deng et al. (2023) [2].

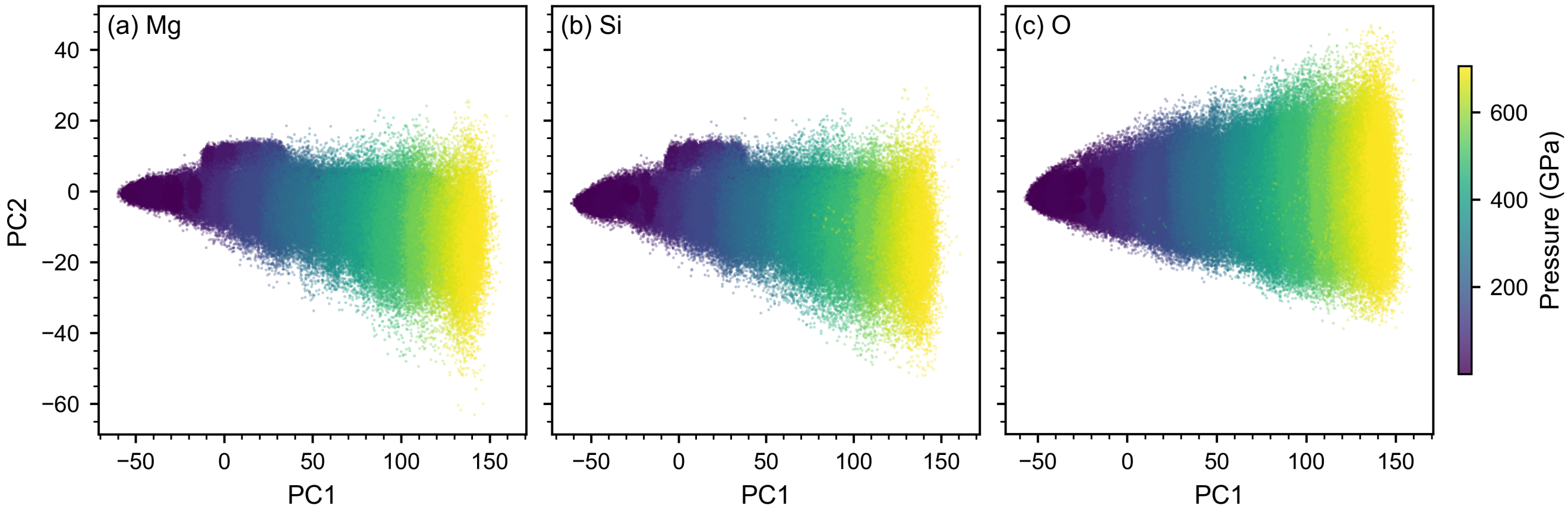

Figure S2. PCA projections of SOAP vectors for (a) Mg-, (b) Si-, and (c) O-centered local atomic environments in MgSiO3 polymorphs and liquid, colored by pressure. As pressure and density are correlated, there exists a correlation between PC1 and pressure. However, it is not as uniform as for density (see Figure 3) because crystals at the same density as the liquid exist at slightly lower pressure. This suggests that higher pressure liquids are needed to mimic similarly dense local atomic environments of crystals at a given pressure.

Draft

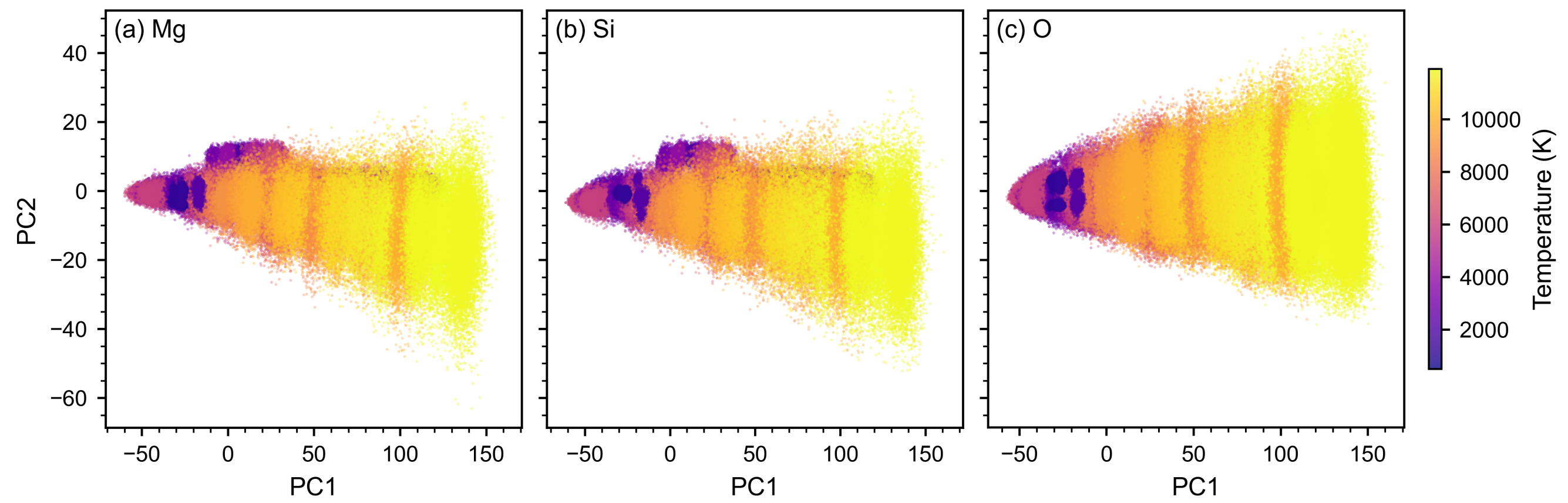

Figure S3. PCA projections of SOAP vectors for (a) Mg-, (b) Si-, and (c) O-centered local atomic environments in MgSiO3 polymorphs and liquid, colored by temperature. Temperature and pressure/density are correlated; thus a correlation between PC1 and temperature exists within each phase but not globally. The solid phases are present at lower temperatures for the same density as the liquid.

Draft

Supplementary Tables

Table S1. Table of percentages of atoms of each crystalline phase found within liquid contours in the PC1-PC2 projection (see Figure 2). For example, the 80% column under "Mg" refers to the fraction of each phase within the 80% contour of liquid points plotted in Figure 2a. Bridgmanite and post-perovskite notably do not lie within most of the liquid points for Mg- and Si-centered local atomic environments, but do for O-centered environments. It should be noted that the liquid contours will be influenced by sampling, where higher sampling of certain temperatures and pressures will concentrate more liquid atoms at those conditions.

	Mg			Si			O		
Phase	80%	95%	99%	80%	95%	99%	80%	95%	99%
brg	2.5	16.2	55.2	3.3	18.3	55.6	100.0	100.0	100.0
ppv	27.0	99.7	100.0	25.8	99.8	100.0	100.0	100.0	100.0
ak	100.0	100.0	100.0	99.9	100.0	100.0	100.0	100.0	100.0
maj	99.9	100.0	100.0	94.3	99.6	100.0	100.0	100.0	100.0
hpcen	100.0	100.0	100.0	99.3	100.0	100.0	96.8	100.0	100.0
ltcpx	100.0	100.0	100.0	100.0	100.0	100.0	100.0	100.0	100.0
oen	99.6	100.0	100.0	100.0	100.0	100.0	100.0	100.0	100.0
proto	99.3	100.0	100.0	100.0	100.0	100.0	100.0	100.0	100.0

Dra

Table S2. Table of correlations for each evaluated descriptor with PC2 for Mg-, Si-, and O-centered environments. Correlations are described with Pearson r and Spearman ρ. Correlations are ordered from most to least correlated via Spearman ρ. Each species is correlated with multiple structural parameters, indicating that SOAP captures a variety of structural information that a single structural parameter cannot capture alone.

Atomic center	Local structural descriptor	Pearson r	Spearman ρ
Mg	Mg-O bond-length standard deviation	-0.40	-0.40
	O neighbors within 6 Å	-0.38	-0.29
	Total neighbors within 6 Å	-0.36	-0.28
	Mean Mg-O bond length	0.28	0.25
	Mg-O coordination number	-0.12	-0.03
Si	Si-O bond-length standard deviation	-0.54	-0.57
	Mean O-Si-O angle	0.44	0.49
	O-Si-O angle standard deviation	0.09	0.38
	O neighbors within 6 Å	-0.37	-0.30
	Total neighbors within 6 Å	-0.32	-0.26
	Mean Si-O bond length	-0.13	-0.16
	Si-O coordination number	-0.22	-0.12
O	O-Mg coordination number	0.72	0.67
	O-Si coordination number	-0.68	-0.60
	Si-O-Si angle standard deviation	-0.52	-0.50
	Total cation coordination number	0.28	0.28
	Mean Si-O-Si angle	0.01	-0.06
	Mg neighbors within 6 Å	-0.00	0.03
	Total neighbors within 6 Å	0.02	0.03
	Si neighbors within 6 Å	0.03	0.02

References

1. T. Duffy, N. Madhusudhan, and K.K.M. Lee. Mineralogy of Super-Earth Planets. In *Treatise on Geophysics*, pages 149–178. Elsevier, 2015.
2. Jie Deng, Haiyang Niu, Junwei Hu, Mingyi Chen, and Lars Stixrude. Melting of MgSiO3 determined by machine learning potentials. *Physical Review B*, 107(6):064103, February 2023.

Draft
